# Factors Associated with Poor Olfaction and Olfactory Decline in Older Adults in the ARIC Neurocognitive Study

**DOI:** 10.3390/nu15163641

**Published:** 2023-08-19

**Authors:** Srishti Shrestha, Xiaoqian Zhu, Vidyulata Kamath, Kevin J. Sullivan, Jennifer A. Deal, A. Richey Sharrett, Andrea L. C. Schneider, Priya Palta, Rebecca F. Gottesman, B. Gwen Windham, Thomas H. Mosley, Michael E. Griswold, Honglei Chen

**Affiliations:** 1 The Memory Impairment and Neurodegenerative Dementia (MIND) Center, University of Mississippi Medical Center, Jackson, MS 39216, USA; 2 Department of Psychiatry and Behavioral Sciences, Johns Hopkins University School of Medicine, Baltimore, MD 21205, USA; 3 Department of Epidemiology, Johns Hopkins University Bloomberg School of Public Health, Baltimore, MD 21205, USA; 4 Department of Neurology, University of Pennsylvania Perelman School of Medicine, Philadelphia, PA 19104, USA; 5 Department of Biostatistics, Epidemiology, and Informatics, University of Pennsylvania Perelman School of Medicine, Philadelphia, PA 19104, USA; 6 Department of Neurology, University of North Carolina Chapel Hill, Chapel Hill, NC 27599, USA; 7 Stroke Branch, National Institute of Neurological Disorders and Stroke Intramural Research Program, Bethesda, MD 20892, USA; 8 Department of Epidemiology and Biostatistics, Michigan State University, East Lansing, MI 48824, USA

**Keywords:** factors affecting olfaction, olfactory change, ARIC Neurocognitive Study

## Abstract

Olfactory function has significant implications for human health, but few risk factors for olfactory decline have been identified. We examined the factors associated with olfactory status and decline over five years in the Atherosclerosis Risk in Communities (ARIC) Neurocognitive Study. A 12-item odor identification test was used to assess olfaction in 6053 participants in 2011–2013 (ARIC visit 5, mean age: 75.6, 41% male, 23% Black race) and in 3235 participants in 2016–2017 (visit 6). We used Poisson regression models to examine cross-sectional associations of a range of potential factors with the total odor identification errors (mean errors: 2.8 ± 2.4) in visit 5 participants. We used mixed-effect Poisson regression to examine associations with olfactory decline between visits 5 and 6. We also examined associations with visit 5 anosmia prevalence (847 cases, 14%) and incident anosmia between the two visits (510 cases, 16%) using Poisson models. Older age, male sex, lower education, Black race, *APOE ε*4 alleles, and diabetes were associated with higher odor identification errors and higher anosmia prevalence, and greater physical activity and hypertension with better olfaction. Age, male sex, lower education, Black race, *APOE ε*4 allele, and vitamin B_12_ levels were associated with incident anosmia over 5 years. Older age was associated with faster olfactory decline. Future studies with longer follow-ups are warranted.

## 1. Introduction

Olfactory impairment affects approximately one-fourth of older adults [1], with prevalence increasing with age up to 63% among individuals aged ≥ 80 years [2]. Although highly prevalent, it is often under-recognized as people tend to be unaware of their olfactory functioning [3]. Olfactory loss can occur from sudden or gradual damage to peripheral olfactory structures (e.g., the olfactory epithelium), the olfactory bulb, or central brain structures responsible for olfactory processing (e.g., the entorhinal cortex). Although generally underappreciated, olfaction has significant implications for human health. Poor olfaction affects multiple aspects of everyday life, including nutrition, safety, social relationships, and mental health, diminishing the overall quality of life [4,5,6]. Furthermore, central olfactory structures are one of the earliest brain structures to be affected in neurodegenerative diseases, including Alzheimer’s disease and Parkinson’s disease, resulting in very early loss of olfactory ability years before the onset of clinical symptoms [7,8]. Olfactory impairment has also been shown to be associated with higher mortality, frailty, and physical function decline, suggesting broader health implications beyond its established associations with neurodegenerative diseases [9,10].

While acquired causes of acute olfactory loss, such as viral infections, traumatic brain injuries, rhinosinusitis, and olfactory structural abnormalities, have been well documented [11,12], few other risk factors of olfactory decline over several years have been identified. In older adults, the prevalence of olfactory impairment increases with age and is about two times as high in men as in women and similarly elevated in Black and White individuals [2,11,13,14]. Studies have also linked smoking [2,15], cardiovascular factors [16,17], diabetes [17], lower physical activity [18], and systemic inflammatory markers [19] with poor olfaction, but findings have been sparse and inconsistent [13,20,21,22,23]. Furthermore, the existing evidence on determinants of olfactory impairment mostly comes from cross-sectional studies [13,23,24,25,26], and only a few prospective studies have investigated the factors associated with longitudinal changes in olfactory function [17,21,22]. Furthermore, while olfactory impairment can affect future nutritional status by altering food perception and dietary behaviors, optimal nutritional status is also important for normal olfaction, as it can influence neuronal health through mechanisms such as neuroinflammation, oxidative stress, and other vascular- and chronic-disease-mediated effects [27,28]. Yet, very few studies have evaluated the associations between nutritional factors and olfaction [26,29,30,31].

Here, we sought to identify the factors associated with olfactory status as well as its change over time in the Atherosclerosis Risk in Communities (ARIC) Neurocognitive Study. Previously, an investigation used ARIC study data to examine cross-sectional associations of a few sociodemographic, behavioral, and cardiovascular risk factors with anosmia prevalence [23]. Here, we extend the prior investigation by examining comprehensive factors, including additional cardiovascular, inflammatory, and nutritional factors that were not previously examined, and conduct new analyses for incident anosmia and 5-year olfactory change.

## 2. Materials and Methods

### 2.1. Study Population

The ARIC study is a prospective cohort study of 15,792 community-dwelling adults (aged 45–64 years at enrollment in 1987–1989) from four US communities: Washington County, Maryland; Forsyth County, North Carolina; Jackson, Mississippi; and suburbs of Minneapolis, Minnesota [32,33]. ARIC participants have undergone multiple comprehensive in-person examinations and are followed up through hospitalization and mortality surveillance. Olfaction was assessed at the fifth (visit 5, 2011–2013) and the sixth (visit 6, 2016–2017) in-person exams (as a part of the ARIC Neurocognitive Study [34]), which were attended by 6538 and 4003 participants, respectively. The current analysis includes participants who completed the visit 5 exam.

After excluding visit 5 participants without olfaction data (n = 445), those other than of Black or White race (n = 15, due to small sample size), and Black participants from the Maryland and Washington sites (n = 25, due to small sample size), we had 6053 participants eligible for our analysis. Of these, 3238 participants completed a second olfaction test at visit 6. The primary analyses included all eligible participants but, in a sensitivity analysis, we excluded prevalent dementia (n = 247) and Parkinson’s disease (n = 33). The study was approved by institutional review boards at all participating study centers, and all participants provided written informed consent.

### 2.2. Olfaction

We used the 12-item Sniffin’ Sticks odor identification test to assess participants’ olfaction [35,36]. This test is commonly used in clinical and epidemiological studies to screen for olfaction impairment. Participants were asked to smell 12 odorants, one at a time, presented in a felt-tip pen, and then pick the correct descriptor from four options. The target odorants were banana, cinnamon, cloves, coffee, fish, leather, lemon, licorice, orange, peppermint, pineapple, and rose. For each correct response, one point was given, with a total possible score of 12. For our primary analysis, we used the total number of odorants incorrectly identified as our continuous analytic outcome. Furthermore, we also examined associations with the categorical outcome anosmia (defined as any participant with a score ≤ 6) [36].

### 2.3. Potential Determinants of Olfaction

Based on the prior literature, we considered sociodemographic (age, sex, race, study site, and education), behavioral (smoking, alcohol consumption, and physical activity), vascular and/or metabolic (body mass index (BMI), serum total cholesterol, diabetes, hypertension status, coronary heart disease, and myocardial infarction), genetic (apolipoprotein E gene epsilon 4 allele (*APOE ε*4)), inflammatory (C-reactive protein), blood hemoglobin, and nutritional factor (serum vitamin B_12_) as the potential risk factors of olfaction [17,18,19,20,21,22]. With the exception of sex, race, study site, and education, which were obtained from the visit 1 exam, other risk factors data were obtained from the visit 5 exam.

Information on sex, race, study site, and education (<high school, high school or equivalent certificate/vocational training, and >high school) was self-reported. Study site and race (site–race) were jointly examined as Washington–White, Forsyth–Black, Forsyth–White, Jackson–Black, and Minneapolis–White due to site–race aliasing. Smoking (current, former, and never) and alcohol consumption status (current, former, and never) were collected using questionnaires. The Modified Baecke Physical Activity Questionnaire was used to obtain information on physical activities including their frequency and duration. The overall physical activity information was expressed as the total metabolic equivalent of task (MET) minutes per week (i.e., energy expenditure per week); details are described elsewhere [37].

BMI (kg/m^2^) was obtained from measured height and weight and examined as a continuous as well as a categorical variable (normal < 25, overweight 25.0–<30, and obese ≥ 30). Serum total cholesterol (mg/dL) was measured using enzymatic methods [38]. Diabetes was defined as fasting glucose ≥ 126 mg/dL or nonfasting glucose ≥ 200 mg/dL, self-report of physician’s diagnosis, or antidiabetic medication use. Hypertension was defined as systolic blood pressure ≥ 140 mm Hg, diastolic blood pressure ≥ 90 mm Hg, or antihypertensive medication use. We also performed a sensitivity analysis with hypertension categorized as normal, prehypertension (defined as systolic blood pressure ≥ 120 and <140 mmHg), and hypertension. History of coronary heart disease, stroke, and myocardial infarction was based on self-reports at baseline and then adjudicated through medical records during the course of the study. The *APOE ε*4 polymorphisms were genotyped using the TaqMan assay (Applied Biosystems, Foster City, CA, USA).

High-sensitivity C-reactive protein, a nonspecific marker of systemic inflammation, was measured in visit 5 samples using an immunoturbidimetric assay on the Beckman Coulter Olympus AU400e analyzer [38]. Serum vitamin B_12_ levels were measured in visit 5 samples using a direct chemiluminescent competitive immunoassay method, and hemoglobin levels were measured in visit 5 whole blood using automated hematology analyzers [38]. Anemia was defined as hemoglobin levels lower than 13 g/dL for men and 12 g/dL for women [39].

### 2.4. Statistical Analysis

We used Poisson regression models to examine the cross-sectional associations of potential risk factors with the number of odor identification errors (i.e., olfaction status at visit 5) and estimated “rate” ratios (RRs) (i.e., the ratio of expected error counts interpreted as the percentage difference in the scores) and 95% confidence intervals (CIs). We used mixed-effect Poisson regression models with random intercepts to examine the associations between these factors and the change in the number of odor identification errors from visit 5 to visit 6. We used an interaction term between the exposure of interest and time to estimate its association with olfactory decline, which we presented as the ratio of “rate” ratio (RRR). In sensitivity analyses, to account for the potential floor effect, we excluded individuals with anosmia at visit 5 and repeated the mixed-effect Poisson models. Covariates in the model varied depending on the exposure of interest under analysis. For example, for site–race as the exposure of interest, we adjusted for age, sex, education, cigarette smoking, and alcohol consumption, and for diabetes as the exposure, we adjusted for age, sex, site–race, education, cigarette smoking, alcohol consumption, BMI, physical activity, total cholesterol, and hypertension.

We also examined the associations of these factors with visit 5 anosmia prevalence using Poisson regression to estimate prevalence ratios (PRs) (n = 6053) although some of these factors have been analyzed in a previous pooled data publication [23]. Furthermore, among those who completed olfactory assessments at both visit 5 and visit 6 and did not have anosmia at visit 5 (n = 2977), we used Poisson regression to estimate the risk ratio (RR) for associations with “incident” anosmia at visit 6. We also repeated analyses for some factors by incorporating the inverse probability of attrition weights to correct for potential bias due to selective attrition from visit 5 to visit 6 as sensitivity analyses.

Furthermore, given the prior findings suggesting racial differences in olfaction [13,14], we also report the associations of these factors with olfaction for White and Black races separately. We used Stata/SE version 16.0 (StataCorp LLC, College Station, TX, USA) for statistical analyses; *p*-value (two-sided) ≤ 0.05 was considered statistically significant.

## 3. Results

Participants’ mean ± SD age at visit 5 was 75.6 ± 5.2 years. About 41% were male, 23% were Black race, and 44% had education higher than high school (Table 1). The participants correctly identified 9.2± 2.4 and 8.9 ± 2.4 odorants on average (the number of errors: 2.8 ± 2.4 and 3.1 ± 2.4) at visit 5 and visit 6, respectively (Table 1, Tables S1 and S2). About 14% (847 of 6053) had anosmia at visit 5 and 16% (510 of 3238) at visit 6. Of the 3238 who completed the olfactory tests at both visits, 261 (8.0%) had anosmia at visit 5. Of the 2977 individuals without anosmia at visit 5, 317 (10.7%) developed anosmia at visit 6. Those who did not attend the visit 6 exam tended to be older and diabetic and were less educated than those who attended the visit 6 exam (Table S2).

In the cross-sectional analysis examining the associations with the number of odor identification errors at visit 5, older age, male sex, lower education, Black race (from Forsyth or Jackson), current or former smoking, any *APOE ε*4 (≥1 allele) carrier, diabetes, and anemia were significantly associated with worse olfactory performance (Table 2). For example, 10-year higher (older) age and male sex were associated with 39% (RR: 1.39 (1.34, 1.43)) and 32% (1.32 (95% CI: 1.26, 1.38)) higher number of error in odor identification, respectively. Compared with White participants in the Washington center, Black participants from the Jackson and Forsyth centers had 64% higher (RR: 1.64 (95% CI: 1.54, 1.74)) and 31% higher (1.31 (95% CI: 1.08, 1.59)) number of error, respectively. For instance, this RR of 1.64% or 64% higher errors can be interpreted as follows: compared with Washington–White participants who made on average three errors in the olfaction test, Jackson–Black participants made five errors (two additional errors) on average. Current drinking, higher physical activity, and hypertension were each associated with lower number of error in odor identification. When hypertension was examined as a three-level categorical variable, only hypertension, but not prehypertension, was associated with olfaction (RR: 0.91 (95% CI: 0.85, 0.98)). Given the inverse association with hypertension, we also examined potential heterogeneity in the association with antihypertensive medication use, but we did not find any interaction (*p*-value for the interaction term: 0.53).

In race-specific analysis, for most of the factors, the associations were similar between White and Black participants (Table S3), except that current smoking was more strongly associated with olfaction only among Whites, and higher BMI was associated with better olfaction only among Blacks. Statistical interaction was significant for concurrent BMI (*p*-value for the interaction term = 0.001) but not significant for smoking (≥0.05).

In the analyses examining changes in continuous olfactory performance from visit 5 to visit 6, we observed that older age was associated with modestly faster olfactory decline, and male sex and Black participants from the Jackson center were associated with slower decline (Table 2). For example, the RRR for 10-year higher age was 1.01 (95% CI: 1.00, 1.02). The results were similar when we excluded individuals with Parkinson’s disease or dementia at visit 5 (Table S4), but only age (RRR: 1.02 (95% CI: 1.01, 1.03) per 10-year higher) was associated with faster olfactory decline after excluding individuals with prevalent anosmia at visit 5 (Table S5).

In the analyses examining anosmia, older age, male sex, and education < high school, Black participants from Forsyth and Jackson, and *APOE ε*4 carrier (≥1 allele) were significantly associated with higher anosmia prevalence at visit 5 (Table 3). BMI ≥ 30, physical activity, and hypertension were significantly associated with lower anosmia prevalence. Unlike odor identification errors as the outcome, alcohol consumption, smoking status, and anemia/hemoglobin were not significantly associated with anosmia prevalence. In the incident anosmia analysis, older age (RR: 2.12 (1.70, 2.65) per 10-year higher); male sex (RR: 1.41 (95% CI: 1.12, 1.76)); lower education (high school: 1.48 (95% CI:1.16, 1.89) and <high school: 1.53 (95% CI:1.10, 2.13) compared with >high school); Forsyth–Black (2.30 (95% CI: 1.12, 4.72)), Forsyth–White (1.46 (95% CI: 1.05, 2.02)), and Jackson–Black (2.51 (95% CI:1.82, 3.45)), each compared with Washington–White; *APOE ε*4 carrier (1.33 (95% CI: 1.05, 1.68)); and history of myocardial infarction (1.55 (95% CI: 1.00, 2.40)) were associated with elevated risk of anosmia at visit 6, and higher serum vitamin B_12_ (0.71 (95% CI: 0.51, 0.98) per 1 SD higher) was associated with lower risk. The incident anosmia analyses for some factors that incorporated the inverse probability of attrition weights were similar to the primary analyses (Table S6). There were no consistent racial differences in prevalent and incident anosmia analyses except that education was more strongly associated with prevalent and incident anosmia in Black participants than in White participants; the interaction term between race and education was statistically significant for prevalent (*p*-value for the interaction term ≤ 0.05) but not for incident anosmia (Table S7).

## 4. Discussion

In this sample of community-dwelling adults, we found that older age, male sex, lower education, Black race, and *APOE ε*4 allele were cross-sectionally associated with poor olfactory status, including anosmia. Furthermore, current cigarette smoking status, diabetes, and anemia were cross-sectionally associated with higher odor identification error, and current alcohol consumption, higher physical activity, and hypertension were associated with fewer errors, although only physical activity and hypertension showed associations with anosmia prevalence. Only older age was associated with faster subject-specific olfactory decline. On the other hand, older age, male sex, lower education, Black race, and *APOE ε*4 allele were associated with higher incident anosmia risk over 5 years.

Despite a high prevalence of poor olfaction in older adults and its potential broad implications on health [9], we know little about potential risk factors for age-related olfaction loss in older adults. Prospective studies on risk factors of olfaction loss are particularly rare; these were mainly based on the Epidemiology of Hearing Loss Study (EHLS) [18,22], the Beaver Dam Offspring Study (BOSS) [16,21], and the Swedish National Study on Aging and Care in Kungsholmen (SNAC-K) cohorts [15,17], all predominantly White populations. These primarily evaluated risk factors in relation to olfactory impairment incidence, and, to our knowledge, only the SNAC-K study has comprehensively examined the associations of risk factors with subject-specific longitudinal change in olfaction performance [17]. The overall study findings have been largely inconclusive, which could, in part, be attributed to the difference in the timing of the exposure and olfaction assessments (i.e., middle-aged vs. older groups); instruments used to assess olfaction (for example, an eight-item test used in the EHLS and BOSS vs. a 16-item test in the SNAC-K study); and follow-up durations in prospective investigations.

In our study, older age was the only factor consistently associated with olfaction, including longitudinal decline. Among all the factors, the association of age with poor olfactory performance is also the most consistent finding across studies [2,17,21,23,24]. Aging individuals may experience greater functional and pathological alterations in the olfactory regions due to wide-ranging factors, resulting in diminished olfactory ability [40,41]. For example, chronic inflammatory nasal conditions and long-term exposure to airborne environmental toxicants may affect peripheral olfaction, and aging-associated neurodegenerative changes within certain brain structures may affect central olfactory processing. We found that male sex and lower education were associated with poor olfaction in cross-sectional analyses and with incident anosmia, but not with olfactory decline. These findings also align with prior studies showing their associations with an elevated risk of olfactory impairment in cross-sectional [2,17,23,24] and prospective investigations [21,22], but not with decline [17]. The potential explanations for sex differences in olfactory ability include sex-hormone-mediated effects on the olfactory system, differences in underlying cognitive/semantic abilities between men and women, and greater hazardous occupational exposure in men [42]. Likewise, educational attainment influences cognition and other lifestyle and occupational exposures that are linked with olfaction.

Compared with White participants from Washington, Maryland, Black participants from Jackson, Mississippi, and Forsyth, North Carolina, had higher anosmia prevalence, consistent with prior cross-sectional reports suggesting worse odor identification ability in Blacks than in Whites [13,23,43,44]. While participants from Jackson and Forsyth overall also showed higher risks of anosmia at visit 6, we did not see faster rates of olfactory decline in these groups; these findings were comparable to one US-based study that found higher odds of poor performance in the future but not greater mean decline in Black participants than in White participants [14]. The higher burden of olfactory impairment in the Black vs. in White race is likely due to systemic socioeconomic and environmental inequities. Black individuals, compared with their White counterparts, tend to have lower education, live in poor housing conditions and areas with heavy pollution (for example, areas with poor air quality and near contaminated sites), and have more hazardous jobs [44,45,46], which are associated with olfactory impairment [47]. Furthermore, Black older adults experience a disproportionately higher burden of cognitive impairment, including dementia [48], for which olfactory impairment is an early symptom; this may also partly explain the racial differences in olfactory abilities. It has also been suggested that Blacks perform worse in odor identification tests due to less familiarity with the odorants used, although the prior ARIC investigation found that Black participants performed consistently worse than White participants in item-wise test performance comparisons, suggesting otherwise [23]. In the ARIC study, due to its study design, we cannot rule out that the observed site–race associations with olfaction are mainly due to racial disparities unique to ARIC sites. When we performed the analyses separately for Whites and Blacks, we did not find consistent race-specific associations, except that education was more strongly associated with prevalent and incident anosmia in Black participants compared with White participants. Future studies are warranted to shed additional light on the association between race and olfaction.

As with race, we observed that the *APOE ε*4 carrier status was associated with poor olfaction at visit 5 and elevated anosmia risk at visit 6, but not with longitudinal olfactory decline. The *APOE ε*4 allele has been linked with elevated tau and amyloid pathology and atrophy in the medial temporal lobe regions (the brain structures implicated in olfactory processing and early Alzheimer’s disease) [8,49,50], even in cognitively normal individuals [49]. Individuals carrying at least one *ε4* allele have been shown to have poor odor identification ability and faster rate of olfactory decline in prior studies, although not always [15,17,23,51,52,53]. For example, in the SNAC-K study, the *APOE ε*4 carrier status was associated with a 12-year olfactory decline [17]. In the same cohort with 6-year follow-up data, the *APOE ε*4 carrier status was associated with elevated odds of future olfactory impairment [15]. In another study, *APOE ε*4 homozygosity was associated with a 10-year decline only in middle-aged participants (45–60 years) but not in older participants (aged ≥ 65 years), suggesting that *APOE ε*4-associated decline may start in midlife [54].

Current and former smokers at visit 5 had lower olfactory scores, but smoking status was not associated with anosmia prevalence or olfactory decline. Cigarette smoke, due to its irritant properties as well as neurotoxic constituents (e.g., cadmium and polycyclic aromatic hydrocarbons), can affect peripheral olfaction through inflammation and direct neuronal damage. Furthermore, these neurotoxicants can directly enter the brain by bypassing the blood–brain barrier and affect central olfactory structures. Prior studies have linked current smokers with higher prevalence and incidence of anosmia, although not consistently [15,18,21,22,23,24,55], whereas former smokers have mostly shown no association [55], leading to the notion that smoking’s effect on olfaction may be reversible. In the ARIC study, there were only a few current smokers at visit 5, and most of the former smokers reported quitting prior to visit 1, which may explain the limited associations observed in the study.

Furthermore, while we found significant associations between physical activity and other cardiovascular risk factors and olfaction in cross-sectional examinations, the directions of the associations were not consistent across the factors. For example, lower physical activity and diabetes were associated with poor olfactory status, whereas BMI ≥ 30 and hypertension were associated with better olfaction. Other than myocardial infarction’s association with incident anosmia, no cardiovascular factors were associated with anosmia risk or longitudinal decline. Due to the well-established links between cardiovascular risk factors and brain health, we anticipated that these factors would be associated with olfactory impairment. Cardiovascular factors, including obesity, diabetes, and hypertension, have been linked with cerebrovascular and neurodegenerative changes [56,57,58] and can affect brain structures involved in olfactory processing, leading to olfactory impairment. Diabetes may disrupt olfactory function also through insulin resistance and other hormonal modulation [59]. Consistent with our findings, prior studies have reported cross-sectional associations of lower physical activity and diabetes with olfactory impairment, although prospective study findings have generally been inconsistent [16,17,18,21,22,59]. Prior findings on BMI and olfaction have also been mixed [15,16,17,18,21,22,60]. In this study, we examined BMI as a potential determinant of olfaction and olfactory decline; however, the association between BMI and olfaction is likely bidirectional, as poor olfaction can affect body mass by altering nutritional intake [61], which may explain the positive association between higher BMI and better olfaction in our cross-sectional analysis. Our finding with hypertension was unexpected and could be a chance finding, but it aligns with the SNAC-K study that found a link between hypertension and attenuation in olfactory decline among older participants (aged ≥ 78 years) [17]. In the EHLS, a population-based study in Beaver Dam, Wisconsin (median age: 66 years at baseline olfactory testing), hypertension was not associated with olfaction [18,22], but in the BOSS study involving adult children of the EHLS (mean age: 49 years at baseline olfactory testing), hypertension was associated with higher incidence of 5-year olfactory impairment and antihypertension medication use in women (but not hypertension) with 10-year olfactory impairment [16,21]. However, these factors were examined as covariates (rather than the exposure of interest) in the analyses that examined subclinical atherosclerosis markers and environmental risk factors as the primary exposures of interest. The disparate associations seen across the studies could also be due to differences in the timing of cardiovascular risk factors and olfaction measurements. Further prospective studies with long-term follow-ups are warranted to provide more insights into this aspect. In the ARIC study, olfaction was measured at a relatively older age (mean age: 75.6 ± 5.8). Although the study collected comprehensive data on cardiovascular factors from midlife through late life, due to a lack of midlife (baseline) olfaction data, we were limited in capturing olfactory change occurring during that period and thus in providing a complete picture of the associations.

Our findings with vitamin B_12_ and hemoglobin were not robust; for example, higher vitamin B_12_ levels were associated with incident anosmia, and higher blood hemoglobin was associated with lower rates of olfactory errors but not with other analytic olfactory outcomes. We know of only a handful of studies that have linked vitamin B_12_ and anemia with poor olfaction, all cross-sectional in nature and some based on small clinical samples [29,31]. So, due to a paucity of data, the current understanding of the association between nutrition and olfaction is very minimal. Still, the associations of these factors with adverse neurological outcomes, including reduced brain volumes (including of those regions involved in olfactory processing, for example, the hippocampus) [62,63,64], lend some indirect support to the notion that these factors might be associated with olfaction.

Although we found that some factors (e.g., male sex, Black race, and lower education) were associated with incident anosmia, there was no evidence for their association with rates of olfactory decline. In our study, olfaction was assessed at only two time points (3 to 7 years apart, median: 5 years) among older participants (mean age: 75.6). So, for within-person changes, it is likely that the 12-item odor identification test was not sensitive enough to detect the subtle declines in olfaction occurring within this relatively short period. Statistically, greater power is required to detect individual differences in change compared with differences in performance levels. Furthermore, if individuals with higher risk profiles (say, male sex, and black race) start with lower olfactory ability in early life, they may reach a threshold for olfactory impairment earlier even if they experience similar rates of decline (as individuals with lower risk profiles), potentially explaining our discrepant findings. Future studies in younger populations and long-term follow-ups may shed more light on the associations of these factors with olfactory decline.

There are also several other limitations to consider. Olfaction was measured at a somewhat older age when the potential risk factors examined (for example, cardiovascular diseases) were already likely at play, and thus this time window when olfaction was assessed may not have been most relevant. We cannot rule out bias due to selective attrition, as only half of the visit 5 participants attended visit 6, and those least likely to attend were also those mostly to have olfactory problems. We did not examine other olfactory domains (e.g., an odor threshold test) that may have provided a comprehensive understanding of the associations with olfaction. For example, an odor threshold test measures a person’s ability to detect odorants and has been attributed more to peripheral olfaction (due to its minimal cognitive dependency), as compared to the odor identification test that may involve central olfactory processing to a greater extent (although they both share common elements). Finally, we used a one-time measurement of nutritional markers which may not reflect long-term body burden due to day-to-day variation in nutrient intake. Nonetheless, our study is one of the largest studies so far to evaluate a wide range of factors in relation to olfactory decline in a sample of Black and White adults.

In conclusion, we found several determinants of poor olfaction in this cross-sectional analysis. While some of these factors were also associated with future anosmia risk, other than with age, we did not see any association between these factors and the longitudinal decline in olfaction. Future studies, especially in younger populations, with longer follow-ups and more detailed assessments of olfaction, are warranted.

## Figures and Tables

**Table 1:** Visit 5 characteristics of the participants relative to visit 5 anosmia status.

Characteristics	Overall (n = 6053)	No Anosmia (n = 5206)	Anosmia (n = 847)
Olfaction (Total Number of Correct Scores, Mean, SD)	9.19 (2.43)	9.96 (1.50)	4.47 (1.57)
Olfaction (Total Number of Errors, Mean, SD)	2.81 (2.43)	2.04 (1.50)	7.53 (1.57)
Age (Years, Mean, SD)	75.60 (5.18)	75.28 (5.05)	77.57 (5.55)
Male (n, %)	2502 (41%)	2061 (40%)	441 (52%)
Black Race (n, %)	1374 (23%)	1028 (20%)	346 (41%)
Education (n, %)			
<High School	878 (15%)	669 (13%)	209 (25%)
High School	2516 (42%)	2206 (42%)	310 (37%)
>High School	2648 (44%)	2324 (45%)	324 (38%)
Study Site (n, %)			
Forsyth, North Carolina	1360 (22%)	1220 (23%)	140 (17%)
Jackson, Mississippi	1279 (21%)	949 (18%)	330 (39%)
Minneapolis, Minnesota	1735 (29%)	1568 (30%)	167 (20%)
Washington, Maryland	1679 (28%)	1469 (28%)	210 (25%)
Cigarette Smoking (n, %)			
Never Smoker	2261 (42%)	1962 (42%)	299 (42%)
Former Smoker	2799 (52%)	2419 (52%)	380 (53%)
Current Smoker	344 (6%)	303 (6%)	41 (6%)
Alcohol Consumption (n, %)			
Never Drinker	1236 (21%)	1046 (21%)	190 (24%)
Former Drinker	1707 (30%)	1414 (28%)	293 (37%)
Current Drinker	2828 (49%)	2529 (51%)	299 (38%)
*APOE ε*4 status (any *ε4* allele, n, %)	1642 (28%)	1351 (27%)	291 (36%)
BMI (kg/m^2^, Mean, SD)	28.81 (5.74)	28.85 (5.64)	28.52 (6.37)
Physical Activity (Total MET-Min/week, Mean, SD)	767.39 (837.95)	791.31 (847.64)	610.85 (753.52)
Serum Total Cholesterol (mg/dL, Mean, SD)	181.48 (42.01)	182.32 (41.78)	176.21 (43.11)
Diabetes (n, %)	1970 (34%)	1650 (32%)	320 (40%)
Hypertension (n, %)	4464 (74%)	3836 (74%)	628 (76%)
Myocardial Infarction History (n, %)	453 (8%)	376 (8%)	77 (10%)
CHD History (n, %)	884 (15%)	729 (14%)	155 (19%)
Stroke History (n, %)	229 (4%)	177 (3%)	52 (6%)
Serum C-Reactive Protein (mg/L, Mean, SD)	4.22 (8.16)	4.16 (8.19)	4.56 (8.01)
Dementia Status (n, %)			
Normal	4529 (75%)	4076 (78%)	453 (54%)
Mild Cognitive Impairment	1264 (21%)	1028 (20%)	236 (28%)
Dementia	247 (4%)	92 (2%)	155 (18%)
Parkinson’s Disease (n, %)	33 (1%)	15 (1%)	18 (4%)
Serum Vitamin B_12_ (pg/mL, Mean, SD)	658.92 (532.09)	657.15 (557.51)	664.49 (442.82)
Blood Hemoglobin (g/dL, Mean, SD)	13.30 (1.48)	13.32 (1.47)	13.15 (1.51)
Anemia (n, %)	1255 (21%)	1022 (20%)	233 (29%)

Abbreviations: BMI, body mass index; CHD, coronary heart disease; MET, metabolic equivalent of task; SD, standard deviation. Missing values: education, n = 11; smoking status, n = 649; alcohol consumption, n = 282; *APOE ε*4 status, n = 254; BMI, n = 179; physical activity, n = 311; total cholesterol, n = 74; diabetes, n = 174; hypertension, n = 54; myocardial infarction, n = 412; coronary heart disease, n = 104; stroke, n = 10; serum C-reactive protein, n = 76; dementia, n = 13.

**Table 2:** Associations of visit 5 characteristics with visit 5 olfaction status and change in olfaction from visit 5 to visit 6.

	Visit 5 Olfaction	Visit 5 to Visit 6 Olfactory Change
Characteristics	RR (95% CI) ^a^	RRR (95% CI) ^b^
Age (per 10-year higher) ^c^	1.386 (1.343, 1.429)	1.010 (1.000, 1.020)
Male ^c^	1.322 (1.264, 1.383)	0.989 (0.980, 0.999)
Education ^c^		
>High School	Referent	Referent
High School	1.069 (1.017, 1.124)	1.006 (0.996, 1.017)
<High School	1.168 (1.097, 1.245)	0.997 (0.983, 1.011)
Study Site–Race ^c^		
Washington–White	Referent	Referent
Forsyth–Black	1.308 (1.075, 1.592)	1.006 (0.966, 1.047)
Forsyth–White	1.016 (0.946, 1.092)	1.009 (0.994, 1.023)
Jackson–Black	1.637 (1.541, 1.739)	0.985 (0.973, 0.998)
Minneapolis–White	0.978 (0.915, 1.045)	0.992 (0.979, 1.006)
Cigarette Smoking ^c^		
Never Smoker	Referent	Referent
Former Smoker	1.051 (1.000, 1.103)	0.999 (0.989, 1.009)
Current Smoker	1.127 (1.035, 1.227)	1.006 (0.988, 1.025)
Alcohol Consumption ^c^		
Never Drinker	Referent	Referent
Former Drinker	0.980 (0.922, 1.042)	1.000 (0.987, 1.013)
Current Drinker	0.920 (0.862, 0.981)	0.996 (0.983, 1.008)
*APOE ε*4 (any *ε4* allele presence) ^d^	1.123 (1.071, 1.178)	0.997 (0.986, 1.008)
BMI (kg/m^2^, per 1SD) ^e^	0.981 (0.958, 1.005)	1.003 (0.998, 1.008)
Obesity Status (BMI (kg/m^2^)) ^e^		
<25	Referent	Referent
25–<30	0.941 (0.881, 1.005)	1.012 (0.999, 1.025)
≥30	0.920 (0.831, 1.020)	1.012 (0.998, 1.025)
Physical Activity (Total MET-Min/week, per 1SD) ^e^	0.956 (0.933, 0.981)	1.000 (0.996, 1.005)
Serum Total Cholesterol (mg/dL, per 1SD) ^f^	0.977 (0.952, 1.002)	0.999 (0.994, 1.004)
Diabetes ^f^	1.075 (1.023, 1.129)	1.004 (0.994, 1.015)
Hypertension ^f^	0.931 (0.881, 0.983)	1.005 (0.994, 1.015)
Myocardial Infarction History ^g^	0.982 (0.895, 1.077)	1.003 (0.984, 1.023)
CHD History ^g^	1.046 (0.970, 1.129)	0.996 (0.981, 1.011)
Stroke History ^g^	1.037 (0.928, 1.160)	0.986 (0.957, 1.016)
C-Reactive Protein (mg/L, per 1SD) ^h^	1.008 (0.986, 1.030)	0.997 (0.989, 1.005)
Serum Vitamin B_12_ (pg/mL, per 1SD) ^h^	1.003 (0.965, 1.042)	0.995 (0.984, 1.005)
Serum Vitamin B_12_ Categories ^h^		
1st Quartile	Referent	Referent
2nd Quartile	0.991 (0.898, 1.093)	1.001 (0.975, 1.028)
3rd Quartile	1.015 (0.917, 1.124)	0.996 (0.971, 1.023)
4th Quartile	1.037 (0.934, 1.153)	0.989 (0.962, 1.016)
Blood Hemoglobin (g/dL, per 1SD) ^h^	0.974 (0.951, 0.996)	0.999 (0.995, 1.003)
Anemia ^h^	1.074 (1.016, 1.135)	0.994 (0.982, 1.007)

Abbreviations: CHD, coronary heart disease; BMI, body mass index; RR, rate ratio; RRR, ratio of (error) rate ratio; SD, standard deviation. One SD unit: BMI: 5.7 kg/m^2^; physical activity: 838 total MET-Min/week; serum total cholesterol: 42 mg/dL; C-reactive protein: 8.2 mg/L; serum vitamin B12: 532 pg/mL; blood hemoglobin: 1.5 g/dL. ^a^: Obtained from Poisson regression models examining cross-sectional associations between visit 5 factors and the number of total errors in the olfactory test; the error rate ratio (RR) is an exponentiated beta estimate (regression parameter) associated with the exposure of interest. ^b^: Obtained from mixed-effect Poisson regression models with the number of total errors in the olfactory test as the repeated measures; the ratio of error rate ratio (RRR) is an exponentiated beta estimate (regression parameter) associated with the interaction term of time with the exposure of interest. ^c^: Obtained from Model 1: age, sex, site–race, education, cigarette smoking, and alcohol consumption. ^d^: Model 2: adjusted for covariates in Model 1. ^e^: Obtained from Model 3: Model 1 covariates, BMI, and physical activity. ^f^: Obtained from Model 4: Model 3 covariates, total cholesterol, diabetes, and hypertension. ^g^: Obtained from Model 5: Model 4 covariates, myocardial infarction history, CHD, and stroke. ^h^: Model 6: adjusted for covariates in Model 4.

**Table 3:** Associations of visit 5 characteristics with visit 5 anosmia prevalence and visit 6 anosmia risk.

	Visit 5 Anosmia	Visit 6 Anosmia
Characteristics	Prevalence Ratio (95% CI) ^a^	Incidence Risk Ratio (95% CI) ^b^
Age (per 10-year higher) ^c^	1.965 (1.740, 2.219)	2.124 (1.701, 2.651)
Male ^c^	1.689 (1.470, 1.941)	1.405 (1.119, 1.764)
Education ^c^		
>High School	Referent	Referent
High School	1.014 (0.865, 1.189)	1.479 (1.159, 1.888)
<High School	1.302 (1.090, 1.555)	1.531 (1.099, 2.134)
Study Site–Race ^c^		
Washington–White	Referent	Referent
Forsyth–Black	1.719 (1.007, 2.933)	2.296 (1.117, 4.719)
Forsyth–White	0.893 (0.706, 1.129)	1.458 (1.050, 2.024)
Jackson–Black	2.354 (1.978, 2.802)	2.506 (1.824, 3.445)
Minneapolis–White	0.903 (0.730, 1.117)	1.023 (0.732, 1.430)
Cigarette Smoking ^c^		
Never Smoker	Referent	Referent
Former Smoker	0.979 (0.844, 1.135)	1.172 (0.919, 1.494)
Current Smoker	0.895 (0.654, 1.225)	1.448 (0.922, 2.275)
Alcohol Consumption ^c^		
Never Drinker	Referent	Referent
Former Drinker	1.013 (0.844, 1.216)	1.167 (0.859, 1.585)
Current Drinker	0.940 (0.769, 1.150)	0.881 (0.646, 1.201)
*APOE ε*4 (any *ε4* allele presence) ^d^	1.254 (1.088, 1.446)	1.330 (1.053, 1.680)
BMI (kg/m^2^, per 1SD) ^e^	0.922 (0.848, 1.002)	1.021 (0.908, 1.147)
Obesity Status (BMI (kg/m^2^)) ^c^		
<25	Referent	Referent
25–<30	0.865 (0.733, 1.019)	1.131 (0.840, 1.523)
≥30	0.752 (0.624, 0.905)	1.215 (0.888, 1.663)
Physical Activity (Total MET-Min/week, per 1SD) ^e^	0.893 (0.821, 0.973)	0.941 (0.838, 1.056)
Serum Total Cholesterol (mg/dL, per 1SD) ^f^	0.929 (0.855, 1.009)	0.939 (0.831, 1.061)
Diabetes ^f^	1.163 (0.999, 1.355)	1.037 (0.811, 1.327)
Hypertension ^f^	0.830 (0.701, 0.983)	1.050 (0.800, 1.378)
Myocardial Infarction History ^g^	0.959 (0.739, 1.245)	1.550 (1.000, 2.403)
CHD History ^g^	1.177 (0.956, 1.451)	0.785 (0.531, 1.160)
Stroke History ^g^	1.019 (0.755, 1.375)	0.477 (0.194, 1.174)
C-Reactive Protein (mg/L, per 1SD) ^h^	0.990 (0.915, 1.070)	1.036 (0.878, 1.222)
Serum Vitamin B_12_ (pg/mL, per 1SD) ^h^	0.976 (0.886, 1.074)	0.706 (0.510, 0.977)
Serum Vitamin B_12_ Categories ^h^		
1st Quartile	Referent	Referent
2nd Quartile	0.845 (0.647, 1.104)	1.054 (0.646, 1.721)
3rd Quartile	1.027 (0.794, 1.328)	1.049 (0.618, 1.781)
4th Quartile	1.014 (0.779, 1.319)	0.650 (0.326, 1.297)
Blood Hemoglobin (g/dL, per 1SD) ^h^	0.962 (0.896, 1.032)	0.961 (0.873, 1.058)
Anemia ^h^	1.109 (0.940, 1.308)	0.939 (0.704, 1.252)

Abbreviations: BMI, body mass index; CHD, coronary heart disease; SD, standard deviation. One SD unit: BMI: 5.7 kg/m^2^; physical activity: 838 total MET-Min/week; serum total cholesterol: 42 mg/dL; C-reactive protein: 8.2 mg/L; serum vitamin B12: 532 pg/mL; blood hemoglobin: 1.5 g/dL. ^a^: Obtained from regression models examining cross-sectional associations between visit 5 factors and anosmia prevalence among 6053 visit 5 participants. ^b^: Obtained from regression models examining the associations between visit 5 factors and risk of anosmia among participants who attended both visits 5 and 6. ^c^: Obtained from Model 1: age, sex, site–race, education, cigarette smoking, and alcohol consumption. ^d^: Model 2: adjusted for covariates in Model 1. ^e^: Obtained from Model 3: Model 1 covariates, BMI, and physical activity. ^f^: Obtained from Model 4: Model 3 covariates, total cholesterol, diabetes, and hypertension. ^g^: Obtained from Model 5: Model 4 covariates, myocardial infarction history, CHD, and stroke. ^h^: Model 6: adjusted for covariates in Model 4. Note: we repeated Model 1 for incident anosmia analysis by incorporating the inverse probability of attrition weights to correct for potential bias due to selective attrition from visit 5 to visit 6. The results were similar to those without incorporating the inverse probability weights.

## Data Availability

The ARIC study data used for this analysis are available to qualified investigators upon request. Details on data availability and study protocols can be accessed at the ARIC website (https://sites.cscc.unc.edu/aric/).

## Supplementary Materials

The following supporting information can be downloaded at: https://www.mdpi.com/article/10.3390/nu15163641/s1, Table S1: Visit 5 and visit 6 olfaction score (total number of errors, mean (SD)) by visit 5 characteristics; Table S2: Visit 5 characteristics of the participants by visit 6 completion status (n = 6053); Table S3: Associations of visit 5 characteristics with visit 5 olfaction status and change in olfaction from visit 5 to visit 6, stratified by race; Table S4: Associations of visit 5 characteristics with visit 5 olfaction status and change in olfaction from visit 5 to visit 6, excluding those with dementia and Parkinson’s disease at visit 5; Table S5: Associations of visit 5 characteristics with change in olfaction from visit 5 to visit 6, excluding individuals with anosmia at visit 5; Table S6: Associations of visit 5 characteristics with visit 6 anosmia using inverse probability weights; Table S7: Associations of visit 5 characteristics with visit 5 anosmia prevalence and visit 6 anosmia risk stratified by race.
